# Can a Computer-Aided Mass Diagnosis Model Based on Perceptive Features Learned From Quantitative Mammography Radiology Reports Improve Junior Radiologists’ Diagnosis Performance? An Observer Study

**DOI:** 10.3389/fonc.2021.773389

**Published:** 2021-12-17

**Authors:** Zilong He, Yue Li, Weixiong Zeng, Weimin Xu, Jialing Liu, Xiangyuan Ma, Jun Wei, Hui Zeng, Zeyuan Xu, Sina Wang, Chanjuan Wen, Jiefang Wu, Chenya Feng, Mengwei Ma, Genggeng Qin, Yao Lu, Weiguo Chen

**Affiliations:** ^1^ Department of Radiology, Nanfang Hospital, Southern Medical University, Guangzhou, China; ^2^ School of Computer Science and Engineering, Sun Yat-sen University, Guangzhou, China; ^3^ Department of Biomedical Engineering, College of Engineering, Shantou University, Shantou, China; ^4^ Perception Vision Medical Technologies Ltd. Co., Guangzhou, China; ^5^ Guangdong Province Key Laboratory of Computational Science, Sun Yat-sen University, Guangzhou, China

**Keywords:** computer-aided diagnosis, digital mammographic, convolutional neural network, mass lesion, diagnosis performance

## Abstract

Radiologists’ diagnostic capabilities for breast mass lesions depend on their experience. Junior radiologists may underestimate or overestimate Breast Imaging Reporting and Data System (BI-RADS) categories of mass lesions owing to a lack of diagnostic experience. The computer-aided diagnosis (CAD) method assists in improving diagnostic performance by providing a breast mass classification reference to radiologists. This study aims to evaluate the impact of a CAD method based on perceptive features learned from quantitative BI-RADS descriptions on breast mass diagnosis performance. We conducted a retrospective multi-reader multi-case (MRMC) study to assess the perceptive feature-based CAD method. A total of 416 digital mammograms of patients with breast masses were obtained from 2014 through 2017, including 231 benign and 185 malignant masses, from which we randomly selected 214 cases (109 benign, 105 malignant) to train the CAD model for perceptive feature extraction and classification. The remaining 202 cases were enrolled as the test set for evaluation, of which 51 patients (29 benign and 22 malignant) participated in the MRMC study. In the MRMC study, we categorized six radiologists into three groups: junior, middle-senior, and senior. They diagnosed 51 patients with and without support from the CAD model. The BI-RADS category, benign or malignant diagnosis, malignancy probability, and diagnosis time during the two evaluation sessions were recorded. In the MRMC evaluation, the average area under the curve (AUC) of the six radiologists with CAD support was slightly higher than that without support (0.896 vs. 0.850, p = 0.0209). Both average sensitivity and specificity increased (p = 0.0253). Under CAD assistance, junior and middle-senior radiologists adjusted the assessment categories of more BI-RADS 4 cases. The diagnosis time with and without CAD support was comparable for five radiologists. The CAD model improved the radiologists’ diagnostic performance for breast masses without prolonging the diagnosis time and assisted in a better BI-RADS assessment, especially for junior radiologists.

## 1 Introduction

Full-field digital mammography (FFDM) is considered an effective method for breast cancer screening (1, 2). In developed and developing countries, FFDM has become the first option for routine medical screening examinations (3); however, the growing number of women coming forward for screening examinations has resulted in an increasing workload for radiologists. The overall diagnosis time for each patient has exhibited an upward trend, indicating that the radiologists’ work efficiency has decreased (4). Lu et al. (5) reported a decline in work efficiency accompanied by the increasing workload of radiologists based on China’s huge population. Karssemeijer et al. (4) illustrated a positive correlation between the increased workload of radiologists and the demand for breast screening.

Owing to a lack of training, inexperienced radiologists have participated in screening prematurely, resulting in diagnostic inaccuracy and insensitivity of breast cancer, with a resulting increased risk of misdiagnosis and missed diagnosis (6–8). As most breast composition in Chinese women are dense breasts, it further increases the difficulty for inexperienced junior radiologists to recognize the characteristics of breast cancer, especially the margins and shape as the main signs (9–11). Friedewald et al. (12) illustrated that radiologists, especially inexperienced junior radiologists, exhibited a decreased diagnostic sensitivity in dense breasts. Broeders et al. (13) believed that junior radiologists were inexperienced in the characteristics of breast cancer, which led to inaccuracy in Breast Imaging Reporting and Data System (BI-RADS) category evaluation and affected the prognosis of patients.

Computer-aided diagnosis (CAD) systems have been introduced as auxiliary methods to improve radiologists’ diagnostic efficiency. Feature extraction is an important step in breast mass classification. Conventional CAD methods extract several handcrafted features from the region of interest (ROI) to form the feature vector for each mass, which comprises three types of features: intensity, shape, and texture (14). In addition, deep learning technology has recently been used in feature design. Deep learning models learn the latent features directly from the ground truth so that more representative features can be designed (15–17). For example, Jiao et al. (18) used a convolutional neural network (CNN) pretrained on ImageNet as a feature extractor for breast mass in breast cancer diagnosis. Kooi et al. (19). combined deep features and conventional handcrafted features to distinguish true masses from normal breast mammary tissue. Their results showed that the combined feature set performed best in the classification stage.

Handcrafted features are designed based on human experience; however, they are not task-specific and may not be effective in medical imaging analysis. A deep learning model learns features by optimizing weights according to the task objective, but this procedure lacks human experience as a clinical reference. To combine radiologists’ clinical experience and deep learning methods to design appropriate features for mass diagnosis, our study proposed a training scheme that considered BI-RADS descriptions. We extracted the features as perceptive features and established a CAD model. Then, we conducted an observer study to evaluate the effectiveness of this model in assisting radiologists in diagnosis.

Our study aimed to verify if a CAD method based on perceptive features learned from quantitative BI-RADS descriptions can help radiologists improve diagnostic performance for mass lesions in mammography, especially junior radiologists.

## 2 Materials and Methods

To ensure that a deep learning-based model obtains sufficient quantitative ability, we used the BI-RADS characteristic description of masses to train a CNN as a perceptive feature extractor. To realize this goal, description quantification, feature extractor training, and classifier training were required in this study. We conducted an observer study to verify the clinical significance of this model. The detailed steps are as follows.

### 2.1 Dataset and Mammogram Collection

We retrospectively retrieved samples between April 2014 and October 2017 from Nanfang Hospital of Southern Medical University, Guangzhou, Guangdong, China. This retrospective study was approved by the institutional review board (IRB)-approved protocol (code number NFEC-2018-037), and informed consent was waived. Breast masses are one of the most common indications for breast cancer. They are more sensitive and easier to detect or diagnose than calcification, architectural distortion, or asymmetry. Because radiologists of differing seniority have varying universality for the diagnosis and detection of mass lesions, they are more suitable as targets for testing whether CAD models help radiologists improve their diagnostic performance.

This study aimed to establish a CAD model for benign and malignant masses, and each case collected in this study had only one mass in the unilateral breast. The pathological results of each mass were used as the gold standard. To avoid confusion regarding the assignment of BI-RADS categories, we excluded cases with calcification, architectural distortion, or asymmetries in this study. We considered only mass lesions to enable radiologists to better focus on BI-RADS categories and the reliability of CAD model results, so as to evaluate the support of CAD models for junior radiologists.

The contralateral breast was negative and confirmed as BI-RADS category 1 or 2. In addition, all collected cases had bilateral craniocaudal (CC) and mediolateral oblique (MLO) images, clinical medical history, radiology reports, and operative and pathological findings. We also excluded patients with implants, lesions not fully visible, or large lesions occupying almost the entire breast in CC and/or MLO mammograms.

**Figure 1:**
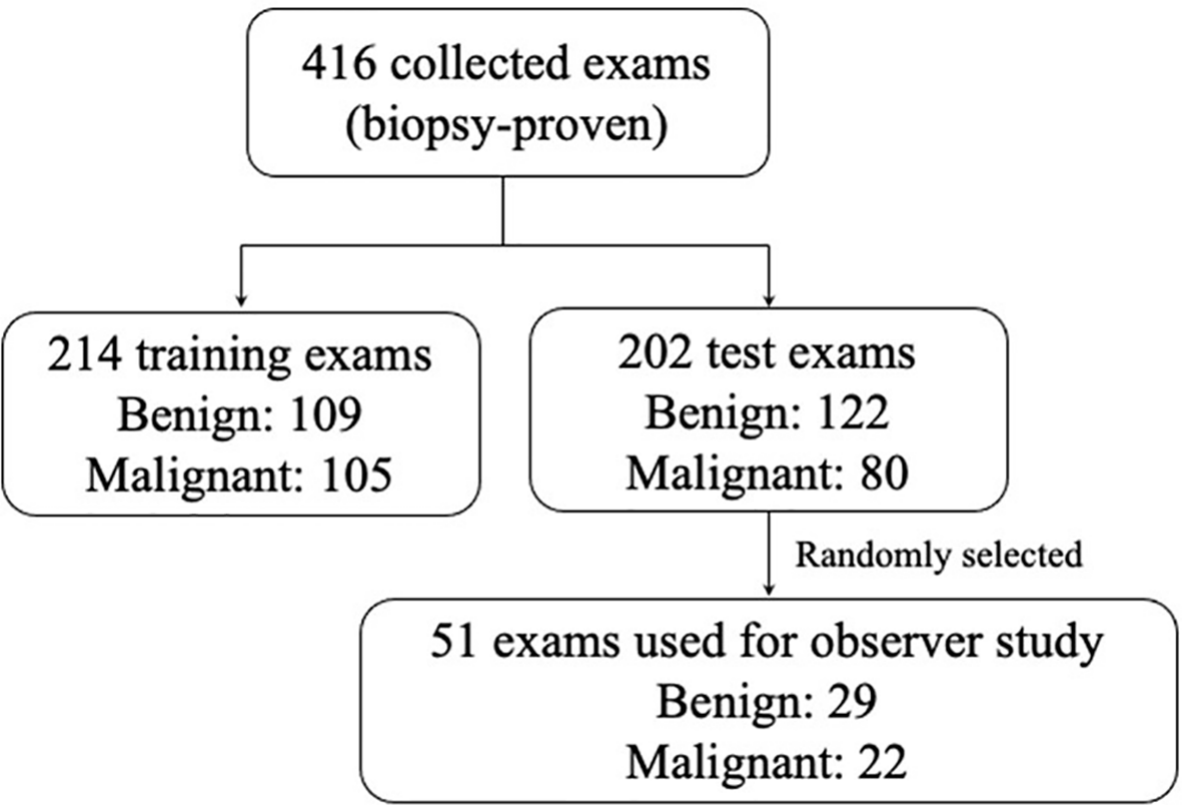
The distribution of the benign and malignant cases obtained in this study. In order to ensure that the study power was better than 0.8, according the reference method, for a ratio of benign and malignant cases of 1.0 in the study and six readers, an evaluation data set of at least 51 cases was needed to be randomly selected.

>In total, 416 cases were obtained, all of which underwent biopsy. Of these, we randomly selected 214 cases (benign 50.9% and malignant 49.1%) as training sets, used to train the feature extractor, and established a mass lesion classification model. The remaining 202 cases (benign 60.4% and malignant 39.6%) were used as independent test sets to evaluate the model, of which 51 cases (benign 56.9% and malignant 43.1%) were randomly selected for the observer study. All the participants were anonymized and represented by a new ID. The specific number of benign and malignant masses is shown in **Figure 1**.

The characteristics of the population and distribution of breast density are shown in **Table 1**. Digital mammography was performed using the Selenia Dimensions System (Hologic, Bedford, MA, USA). The size of each image was 3,328 × 2,560 with a pixel spacing of 0.06 mm. Detailed information regarding these 416 cases is provided in **Appendix A**.

**Table 1:** Characteristics about the age, breast composition, and biopsy results of the population for this study.

Variable	Training set (n = 214)	Test set (n = 202)	51 Cases in observer evaluation
**Patient age (years)**			
Mean	45.64	45.51	46.53
Median	45	45	47
Range	23–73	23–78	27–65
Interquartile range	40–50	40–50	40–51
p-value compared with training set	–	0.8910	0.5441
BI-RADS breast composition^*^			
a	6	5	3
b	23	25	9
c	169	155	35
d	16	17	4
p-value compared with training set	–	0.9268	0.3404

^*^BI-RADS breast composition is defined in the fifth ACR BI-RADS; it includes four categories. “a”: almost entirely fatty; “b”: scattered areas of fibroglandular density; “c”: heterogeneously dense; “d”: extremely dense.

BI-RADS, Breast Imaging Reporting and Data System.

### 2.2 Region of Interest Selection

An experienced radiologist (X. Liao, with 15 years’ experience in digital mammography) marked the ROI areas of all masses using a three-monitor Hologic diagnostic workstation (SecurViewDx, Hologic, MA, USA) on the standard FFDM (both CC and MLO views). Three radiologists (G. G. Qin, L. Zhang, and W. G. Chen, with 15 years of experience each) reviewed the ROI to ensure its suitability. In case of any disagreements regarding the location or shape of a certain mass among these three radiologists, they determined the final ROI area (marked by these radiologists) through majority-voting and discussion until a consensus was reached.

### 2.3 Mass Classification Model

#### 2.3.1 Quantification of the BI-RADS Description

Our CAD model was intended to extract perceptive features and reflect semantic characteristics, such as a human’s visual perception and diagnosis experience. This experience is reflected in radiology reports. Several descriptions, including shape, margins, and density, are defined in the BI-RADS lexicon for mass, which are the main factors for radiologists in diagnosing breast cancer.

Different descriptions have different probabilities for malignant masses. For example, irregular shapes or indistinct margins are correlated with suspicious findings, and oval or circumscribed margins are correlated with benign findings. In perceptive feature design, these descriptions are used as the ground truth to train a regression network. We intend the perceptive features to comprise the feature vector in the last fully connected layer.

**Figure 2:**
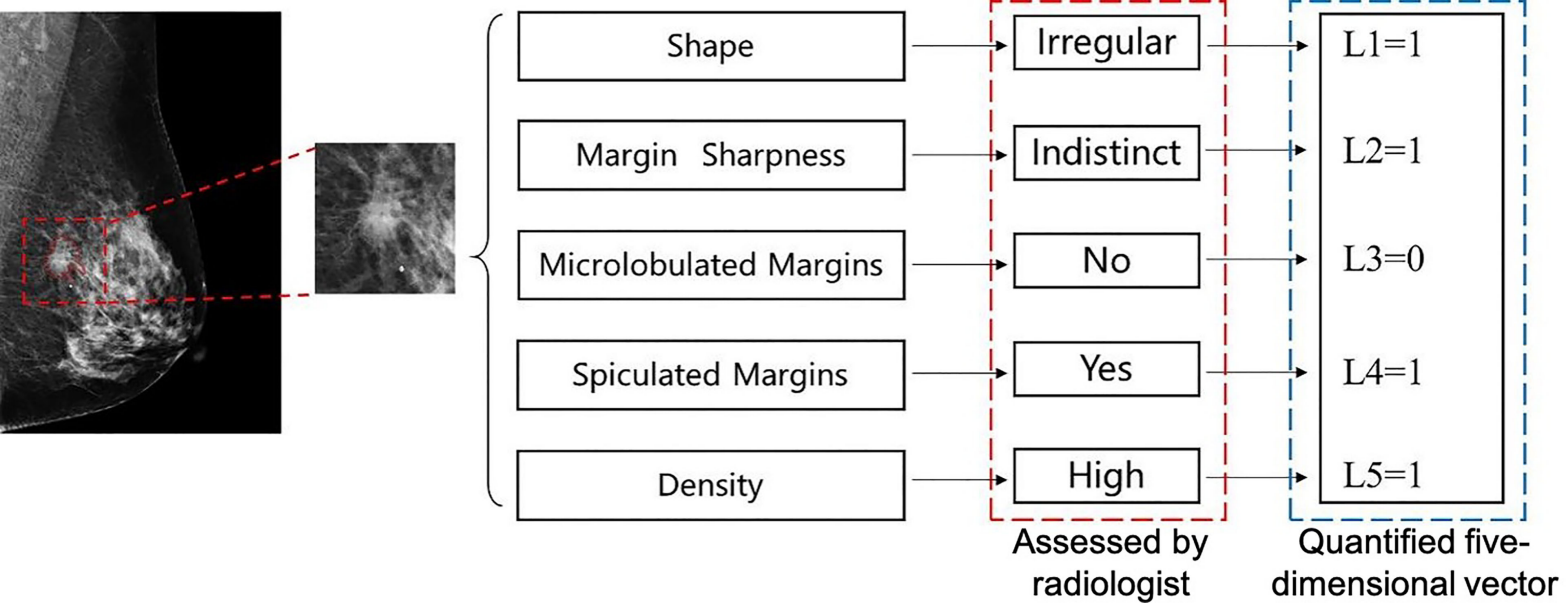
An example of quantification for a malignant mass in mediolateral oblique (MLO)-view full-field digital mammography (FFDM). Five text descriptions assessed by a radiologist as shown in the red box are quantified as corresponding numbers. A five-dimension vector is generated, which is used as the ground truth to train the perceptive feature extractor.

However, the training procedure requires a quantitative ground truth instead of text descriptions; therefore, it was necessary to quantify these descriptions. To correlate the quantification to the classification task, we quantified the descriptions as a malignancy probability. Descriptions of malignant, uncertain, and benign findings were quantified as 1, 0.5, and 0, respectively. Details of the quantification are provided in **Table 2**. For example, if a case has such descriptions in its radiology report: “An *irregular* mass with *obscured* and *microlobulated* margins and *high density* is present,” a five-dimensional vector can be quantified as [1, 0.5, 1, 0, 1] according to these descriptions. The five entries represent the quantification for shape, margin sharpness, microlobulated margins, spiculated margins, and density, respectively. Another example is shown in **Figure 2**.

**Table 2:** Specific quantification for different descriptions summarized from radiology reports of all cases in our study.

Descriptions	Radiologists’ assessment	Quantification
Shape	Oval or round	0
	Irregular	1
Margin sharpness	Circumscribed	0
	Obscured	0.5
	Indistinct	1
Microlobulated margins	No	0
	Yes	1
Spiculated margins	No	0
	Yes	1
Density	Low or fat-containing	0
	Equal	0.5
	High	1

#### 2.3.2 Stage 1: Feature Extractor

The backbone of the feature extractor is a classical CNN VGG16 (20). It is used to classify the class of objects in natural images and plays an important role in CAD. In this study, we made a slight modification to VGG16 to meet our needs.

**Figure 3:**
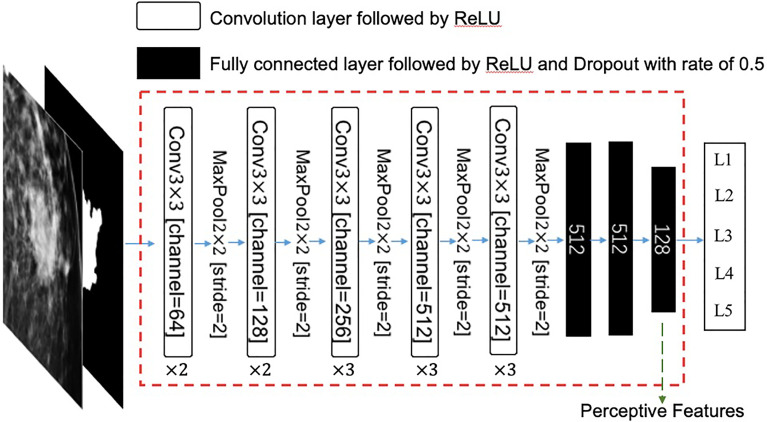
The architecture of this feature extractor. The input is a two-channel tensor, which consists of an original mammography patch and its corresponding mask of region of interest (ROI). The extractor is a modified VGG16 neural network, which consists of 13 convolution layers and three fully connected layers. The last fully connected layer has 128 neurons, which are used as perceptive features in this study. ReLU, rectified linear unit; Conv, convolution.

A two-channel patch with a size of 288 × 288 centered at a mass was used as the input for the network. One channel is an original FFDM, and the other is a binary mask that represents the ROI. The remaining convolution layers, activation functions, and pooling layers are the same as those in the original VGG16 network. Then, three fully connected layers with rectified linear unit (ReLU) activation functions and dropout operations were used to convert this feature map into a feature vector. Finally, the network outputs a five-dimension vector, which represents the predicted quantitative descriptions of the input mass. The specific architecture of this feature extractor is illustrated in **Figure 3**.

The mean square error was used as a loss function. The weights of each layer were initialized randomly according to a standard normal distribution and updated using an Adam optimizer during the training process. Both CC- and MLO-view masses were fed into the same feature extractor. In the training process, the masses of the same patient in the CC-view and MLO-view FFDM shared the same quantitative BI-RADS descriptions.

Since this network is trained using the quantitative BI-RADS descriptions as ground truth, its weights can implicitly represent these descriptions, which are the perception of radiologists when writing the report. Therefore, after the VGG16 network was trained, we discarded its output layer so that the remaining network would output a 128-dimension feature vector and we call these features “perceptive features.” Then, the weights of the remaining network are frozen, and the network is used as the feature extractor in the next stage.

#### 2.3.3 Stage 2: Benign and Malignant Classification

These features were then used to train a classifier that can distinguish between benign and malignant masses. Stepwise regression feature selection and linear discriminant analysis (LDA) classifiers were employed to achieve this goal.

In the training of stepwise regression and LDA, we did not differentiate between masses from CC-view and MLO-view images; that is, a lesion-wise classification model was considered. In the test process, the malignancy probability output by the model from the CC-view and MLO-view images of the same case were averaged for case-wise evaluation.

#### 2.3.4 Model Selection and Test

Ten-fold cross-validation was used. The 214 training cases were randomly divided into 10 folds. In each training time, nine folds were used as the training set, and one fold was used as the validation set. The feature extractor was trained until the loss of both sets plateaued. After 10 repetitions, all folds were used as the validation set once, and 10 trained models were obtained.

During the test process, the 10 trained models were used on the 202 independent test cases, and 10 predicted scores were output for each case.

Model fusion always obtains a better model whose performance exceeds that of each individual. To fuse these 10 trained models, the averaged probability of malignancy (POM) among the 10 models was calculated for each case, which was used in the multi-reader multi-case (MRMC) evaluation.

### 2.4 Observer Evaluation

#### 2.4.1 Multi-Reader Multi-Case Evaluation

We assessed the diagnostic performance both with and without the aid of our model to demonstrate the utility of our model in a real-world setting. Unaided and aided performance was evaluated in a total of 51 cases across two separate sessions with a time interval of more than 15 days.

To avoid individual diagnosis differences at different times for the same radiologist in the same cases, multiple radiologists were involved in this observer study. Six radiologists participated in the study. Two of them were junior radiologists with 2 years of experience (reader 1: CF and reader 2: MM), two of them were middle-seniority radiologists with 4 years of experience (reader 3: ZX and reader 4: SW), and the remaining two radiologists were senior with 6 years of experience (reader 5: JW and reader 6: HZ).

In the first session, each radiologist observed the FFDM of the cases without the model's outputs (unaided evaluation) . Only the original images of each mass with ROI in both the CC-view and MLO-view FFDM were provided. In the second session, we also provided the POM calculated by the model during the diagnosis (aided evaluation). All cases were interpreted using a three-monitor Hologic diagnostic workstation (SecurViewDx, Hologic, MA, USA) . In both sessions, the observers recorded the BI-RADS category, benign or malignant classification, POM (ranging from 0% to 100%), and time consumption (in s) for statistical analysis. Detailed results are recorded in a table presented in **Appendix B**.

Before these two sessions, the six radiologists learned the evaluation criteria and announcements. We used 20 example cases to train the radiologists to perform this process. The radiologists were not informed about history taking, results from other examinations, and palpation.

### 2.5 Statistical Analysis

The p-value for the age difference between the training set and the test set was calculated by unpaired t-test, and the p-value for breast density was calculated by chi-square test.

In the MRMC study, we calculated the area under the receiver operating characteristic (ROC) curve according to the POM assessed by radiologists for each session. ROC curves were obtained by ranking all POMs evaluated by a certain radiologist in a certain case set in ascending order. The true positive rate (TPR) and false positive rate (FPR) were calculated at the probability threshold of each ranked POM. Taking all of the TPRs as coordinates on the y-axis and all of the FPRs as coordinates on the x-axis, the ROC curve was plotted on this case set for this radiologist.

To analyze the significance between two evaluation sessions, Wald or z-test was used to yield a p-value with the null hypothesis that these two sessions had the same areas under the curve (AUCs). To compare the sensitivity and specificity between the two sessions, the assessment results for benign and malignant masses were compared with the biopsy-proven ground truth. A binary-version MRMC analysis was implemented to yield a p-value. The average diagnosis time of each case was calculated for each radiologist in each session, and the paired t-test was used to yield the p-value for the difference between the two sessions.

The “iMRMC,” “ROCR,” and “pROC” packages in R language were used to conduct the statistical analyses in this study.

## 3 Results

### 3.1 Parameter Selection

The learning rate of the Adam optimizer was initialized to 0.0001. We set two decay times with decay gamma of 0.1 at epoch 30 and 60, respectively.

During the training process, we obtained the loss curve of each training and validation set in a 10-fold cross-validation. The validation loss reached the lowest value and plateaued when the training reached the 70th epoch. We also found that the loss curves were similar to those in the other nine cross-validation times. As a result, we fixed the weights of the feature extractor at the 70th epoch of the VGG16 network.

In the analysis, we implemented stepwise regression and an LDA classifier using MATLAB 2018a with default parameters.

### 3.2 Cross-Validation and Independent Test

In the cross-validation, the vertical average AUC value of the validation sets was 0.95, while the maximum value was 0.99, and the minimum value was 0.86. The vertical average ROC curve of 10-fold validation is shown in the **Supplementary Material**.

In the 202 independent test cases, the AUC value was 0.91.

### 3.3 Receiver Operating Characteristic Performance

**Figure 4:**
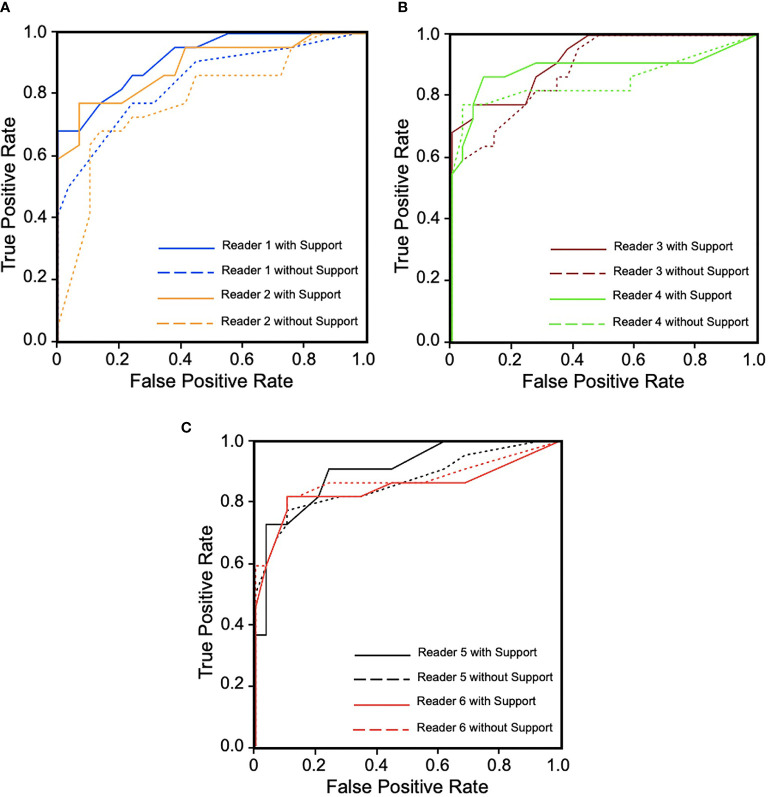
Receiver operating characteristic (ROC) curves for six readers in three groups during diagnosis with and without computer-aided diagnosis (CAD) model support. **(A)** Junior group of readers 1 and 2. **(B)** Middle-seniority group of readers 3 and 4. **(C)** Senior group of readers 5 and 6. We can observe that ROCs show an upward trend more highly and steadily in junior group, but in middle-seniority and senior groups, ROCs are not obviously changed, which has decreased with support in reader 6.

In the MRMC study, readers’ average AUCs increased from 0.850 to 0.896 (p = 0.0209). With support from our model, we found that five of the six radiologists’ AUC scores (reader 1: 0.078, reader 2: 0.109, reader 3: 0.033, reader 4: 0.038, and reader 5: 0.038) increased by an average of 0.059, especially junior radiologists (reader 1 increased 0.078 and reader 2 increased 0.109), but one radiologist’s AUC decreased (one of the senior radiologists, reader 6: -0.022). Detailed specific AUC changes are shown in **Table 3**. The ROC curves in **Figure 4** show that the junior group’s ROCs have an upward trend more highly and steadily, but those in middle-seniority and senior group show no appreciable change.

**Table 3:** The comparisons for specific AUCs for six readers and their averaged AUC in multi-reader multi-case observer study.

Reader	AUC unaided	AUC with model reference	Difference	p-value
1	0.842	0.920	0.078	
2	0.783	0.892	0.109	
3	0.889	0.922	0.033	
4	0.852	0.890	0.038	
5	0.866	0.904	0.038	
6	0.869	0.847	-0.022	
Diagonal average	0.850	0.896	0.046	0.0209

AUC, area under the curve.

### 3.4 Benign and Malignant Evaluation

The sensitivity and specificity of the radiologists in the two sessions are shown in **Table 4**. The sensitivities of all radiologists with model support were higher or equal to those without model support (junior group, increased on average 0.160; middle-seniority group, increased on average 0.046; and senior group, increased on average 0.041). We found that the junior group’s sensitivity increased significantly (reader 1 increased by 0.137 and reader 2 increased by 0.182); however, the sensitivity of the middle-aged and senior groups remained stable (**Table 4**). The specificities of five of the six radiologists were higher than or equal to those without model support (junior group unchanged, middle-seniority group increased on average 0.087, senior group decreased on average 0.035), particularly reader 3: 0.104 and reader 4: 0.069. The specificity of reader 5 decreased by 0.104. PPV and NPV were only slightly increased after aiding by the CAD model (PPV average increased 0.045, NPV average 0.058).

**Table 4:** The difference in sensitivity, specificity, PPV, and NPV in different experience groups with and without model reference.

Sensitivity	Reader	Unaided	Aided	Difference
Group				
Junior	1	0.545	0.682	0.137
	2	0.682	0.864	0.182
Middle-seniority	3	0.773	0.773	0
	4	0.773	0.864	0.091
Senior	5	0.819	0.901	0.082
	6	0.773	0.773	0
**Specificity**				
**Group**	**Reader**	**Unaided**	**Aided**	**Difference**
Junior	1	0.931	0.931	0
	2	0.621	0.621	0
Middle-seniority	3	0.793	0.897	0.104
	4	0.862	0.931	0.069
Senior	5	0.793	0.689	-0.104
	6	0.863	0.897	0.034
**Positive predictive value (PPV)**				
**Group**	**Reader**	**Unaided**	**Aided**	**Difference**
Junior	1	0.857	0.882	0.025
	2	0.577	0.633	0.056
Middle-seniority	3	0.739	0.850	0.111
	4	0.810	0.905	0.095
Senior	5	0.750	0.689	-0.061
	6	0.809	0.850	0.041
**Negative predictive value (NPV)**				
**Group**	**Reader**	**Unaided**	**Aided**	**Difference**
Junior	1	0.729	0.794	0.065
	2	0.720	0.857	0.137
Middle-seniority	3	0.821	0.838	0.017
	4	0.833	0.900	0.067
Senior	5	0.851	0.909	0.058
	6	0.833	0.838	0.005

Binary MRMC analysis showed that the performance improvement was significant (p = 0.0253).

### 3.5 BI-RADS Evaluation

In MRMC evaluation, all readers adjusted the BI-RADS category of partial cases with model support, which focused on BI-RADS 2–4. The BI-RADS 4 category was the most confused and most-adjusted (total changed 87 cases, included 52 increased cases and 35 decreased cases). After model support, more follow-up cases were adjusted for malignancy or need to be focused and recalled for further biopsy. The detailed changes in the BI-RADS are shown in **Table 5**, and more detailed information about the BI-RADS evaluation is provided in **Appendix C**.

**Table 5:** Counts of BI-RADS changes for each reader under supporting by CAD model.

		Junior group		Middle-seniority group		Senior group		Total
		Reader 1	Reader 2	Reader 3	Reader 4	Reader 5	Reader 6	
**Changes**	**Increase**	14	11	11	17	22	5	80
	**Decrease**	4	15	13	5	4	7	48
	**Total**	18	26	24	22	26	12	128
**Compare the revised results with the biopsies**	**Closer**	10	15	13	8	14	7	67
	**Not real matching**	8	11	11	14	12	5	56
**Changed cases’ breast composition**	**a**	1	0	1	0	1	0	3
	**b**	2	7	5	2	6	1	23
	**c**	12	18	16	17	17	10	90
	**d**	3	1	2	3	2	1	12

BI-RADS, Breast Imaging Reporting and Data System.

### 3.6 Diagnosis Time

**Figure 5:**
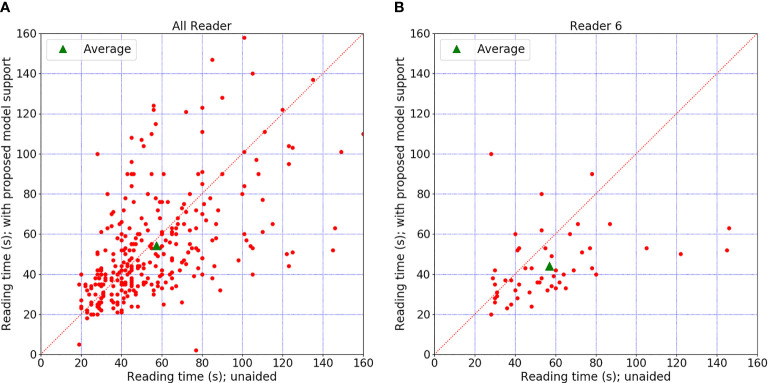
Diagnosis time comparison. **(A)** The time comparison of all readers. **(B)** The time comparison for reader 6, who was the only one who showed an obvious difference between the two sessions. The graph shows differences in diagnosis time per case for all readers. Each red point indicated diagnosis time for a certain case with or without model support. There is no significant change when the point falls on the diagonal. Point above the diagonal indicates diagnosis time increased with model support. Point below the diagonal means the time decreased with model support.

Each radiologist recorded the diagnosis time using timer software with the number of seconds. The average diagnosis times per case for radiologists in these two sessions are shown in **Table 6**. Five of the six radiologists had comparable diagnostic efficiency. Reader 6’s diagnosis time significantly decreased from 56.96 to 43.96 (almost reduced by 22.8%) after involving the CAD support (p = 0.01). Two senior experienced radiologists showed a larger decrease in diagnosis time than the other radiologists. We can observe that the diagnosis time may increase slightly in readers 1–3 and decrease in readers 4–6. This might be because the senior group analyzes the signs of the mass lesion more confidently under the support of the CAD model. **Figure 5** shows the reading time comparison for all readers.

**Table 6:** The mean diagnosis time for radiologists in multi-reader multi-case study.

Reader	Mean time w/o support(s)	Mean time with model support (s)	Difference	p-value	With model increased time cases	With model decreased time cases	With model remained the same time cases
1	55.27	55.51	0.24	0.955	22	29	0
2	80.59	81.18	0.59	0.912	12	38	1
3	63.90	64.24	0.34	0.928	22	27	2
4	45.10	42.59	-2.51	0.378	9	39	3
5	42.35	37.35	-5	0.089	14	36	1
6	56.96	43.96	-13	0.001	19	32	0

## 4 Discussion and Conclusions

In this study, we proposed a deep learning-based perceptive feature extractor for breast mass lesion classification. To evaluate this CAD model, we conducted an observer study to verify whether this CAD model could help radiologists, especially in the junior group. The results showed that this CAD model assisted radiologists in improving diagnostic accuracy and confidence without excessively increasing diagnostic time.

This perceptive feature extractor used quantitative BI-RADS descriptions instead of biopsy-proven results to optimize the weights. This brought benefits to the CAD model, and a total of 128 features were extracted. First, we obtained BI-RADS descriptions from radiologists. When optimizing, human visual perception and clinical experience were integrated into the weights. This provided us with more ideas for interpreting the learned features of CNN. Second, compared to using a CNN directly to establish a CAD model, the feature extractor was first trained and then a classifier was used to complete the diagnosis, which was more consistent with the process of clinical diagnosis.

As summarized in **Table 7**, we compared the diagnostic performance achieved by our model with those of other recently published classic CAD models and deep learning models (21–27). In these studies, researchers used different classifiers for mass classification. A notable exception is the study by Yan et al. (27), who combined dual-view mammogram matching and CMCNet VGG16, similar to the quantification of the BI-RADS description combined with classical CNN VGG16 in our study.

**Table 7:** Diagnostic performance of previous models in classification of masses.

Year	First author	Model	Traning cases	AUC
2015	Ertosun and Rubin (21)	VGG -Net 16	2,250	0.82
2015	Surendiran et al. (22)	A univariate ANOVA discriminant analysis	300	0.93
2016	Sun et al. (23)	4 Convolutional ANN and 1 fully connected layer	840	0.70
2017	Becker et al. (24)	ViDi Red	286	0.81
2020	Agarwal et al. (25)	Faster-RCNN	800	0.90
2020	Boumaraf et al. (26)	BPN	500	0.94
2021	Yan et al. (27)	CMCNet VGG16	586	0.94
2021	Ours	Classical CNN VGG16 based on perceptive features	214	0.91

In the observer study, the AUC scores showed that the radiologists had a higher and better diagnostic performance with CAD model support, which is similar to earlier reported studies (28); however, these studies did not mention the relationship between the radiologist’s experience and the CAD model effects. In our study, readers were divided into junior, middle-seniority, and senior groups according to their experience. The AUC changes in these groups showed that junior radiologists have the largest improvement with the help of the CAD model, which means that the model could provide a more important reference for junior radiologists. It has potential to play a role in junior radiologists’ training.

Furthermore, the diagnostic sensitivities of all radiologists increased when the model was involved, which was similar to that reported by Boumaraf et al. (26). Under CAD model aiding, radiologists not only obtained more information about the likelihood of malignancy but also reduced the breast density effect. This help was significant for junior radiologists during the diagnosis of suspicious malignant cases, which made more cases assessed for malignancy and increased sensitivity. However, for those who are experienced radiologists, such assistance may not make much difference, so the sensitivity (for middle-seniority and senior groups) remained the same, if not slightly higher than that without the model. Meanwhile, support by the CAD model meant that the radiologists could obtain more details from the mass’s shape and sharpness, which decreased the confusion in BI-RADS 4 or other suspicious cases, especially within the middle-seniority group. This means that the CAD model could be a supplement to clinical inexperience, just as we observed with the change in sensitivity and specificity in the study. Except for reader 5, where the extra information from the CAD model was unhelpful, instead adding more confusion, which decreased specificity (29–31). Although PPV and NPV were only slightly increased after aiding by the CAD model, the increase was insufficient to prove the clinical screening significance of the CAD model; however, we still believe that the CAD model could help radiologists in terms of interpretability and clinical confidence.

In addition, the radiologists adjusted the evaluation of the BI-RADS category after the CAD model was involved. BI-RADS 4 categories are the most confusing type, which makes radiologists take a lot of time in the classification and recognition of signs as diagnostic evidence (24). We observed that the radiologists in this study adjusted many BI-RADS 4 cases in the second session (among 128 changed cases, 87 of them were BI-RADS 4 cases). On the other hand, in this adjustment, the ratios of cases whose BI-RADS diagnosis was closer to that of biopsy are 56.55%, 45.20%, and 56.05% for junior, middle-seniority, and senior groups, respectively. The POM score offered by the model helped the radiologist make a better decision in those difficult cases or complex cases, providing more evidence for biopsies. Therefore, radiologists pay more attention to the most suspicious characteristics of mass lesions (32).

Considering the workload of radiologists in daily work, we hope that using a CAD model will not increase the diagnosis time for each patient. Therefore, we proposed a quantification of the BI-RADS description method to establish the CAD model, which would be more interpretable for radiologists. The model outputting the POM for the mass lesion’s likelihood of malignancy would reduce the difficulties in understanding and could be a better auxiliary means for the diagnostic process, spending less time in making a comprehensive judgment, and thus would not increase the workload. This means that a quantification of the BI-RADS description method would be more suitable for the subjective consciousness of junior radiologists, giving them more confidence in the CAD model result, possibly decreasing the diagnostic burden (33).

Our study has some limitations. First, our model is not an end-to-end CAD system. To verify the CAD model’s correlation associated with radiologists’ experience, we focused only on mass, which is an uncomprehensive system for all the signs in breast lesions. Second, compared with other similar studies, where an average of 16.4 radiologists participated in the reader study (34–38), the number of readers in MRMC was relatively small in our study. Moreover, this study did not set two replicate experiments to verify the agreements for the same radiologist in different reading times. Third, this was a single-center study. Different regions have different population distributions. We did not explore if this CAD model could be applied to other populations outside our institution, which might not have sufficient universality.

In future work, more solutions will be proposed to address these limitations shown above. First, we will explore the automatic detection and segmentation models for breast mass. Although there are many CAD models about mass detection and segmentation, they are not fully appropriate for the cases with dense breast. Thus, our model will focus on the cases with dense breast, which are common in China. Combining the detection, segmentation, and our classification model proposed in this study, an end-to-end CAD model can be established for China’s poplution. Second, more radiologists will participate in our reader study. The more readers who participate, the more solid the conclusion will be. Third, a multicenter study should be considered in the future. Domain adaptation technology will be used to reduce the gap of data distribution between data centers.

In general, the perceptive feature-based CAD model improved the radiologists’ diagnostic performance for breast masses without improving the diagnostic time and assisted junior radiologists with better assignment in BI-RADS 4 categories. This illustrates that the model has potential to train junior radiologists and help them improve the diagnosis accuracy for breast mass.

## Data Availability Statement

The datasets presented in this article are not readily available because the limitations of patient privacy policy and the Medical Ethics Committee of Nanfang Hospital. Requests to access the datasets should be directed to the corresponding author.

## Ethics Statement

The studies involving human participants were reviewed and approved by the Medical Ethics Committee of Nanfang Hospital. The code number of clinical ethics project is NFEC-2018-037. Also, all participants’ consent was waived by the Medical Ethics Committee of Nanfang Hospital. Written informed consent for participation was not required for this study in accordance with the national legislation and the institutional requirements.

## Author Contributions

ZH collected data, designed the experiments, and wrote the article. YLi wrote the code, implemented the experiments, and wrote the article. WZ and JL were responsible for formatting. WX, CW, HZ, ZX, SW, JWu, CF, and MM participated in the observer study. XM and JWe provided guidance for establishing a deep learning model. WC, YLu, and GQ provided guidance for the entire study, including model design, experiment design, and article writing. All authors contributed to the article and approved the submitted version.

## Funding

This study received partial funding from the National Natural Science Foundation of China Nos. 82171929; the National Key R&D Program of China under Grant Nos. 2018YFC1704206 and 2016YFB0200602, 2019YFC0121903 and 2019YFC0117301; the Fundamental Research Funds for the Central Universities under Grant No. 19LGYJS63; the NSFC under Grant Nos. 81971691, 81801809, 81830052, 81827802, U1811461, and 11401601; the Natural Science Foundation of Guangdong Province, China, under Grant Nos. 2019A1515011168 and 2018A0303130215; the Science and Technology Innovative Project of Guangdong Province under Grant Nos. 2016B030307003, 2015B010110003, and 2015B020233008; the Science and Technology Planning Project of Guangdong Province under Key Grant Nos. 2015B020233002 and 2017B020210001; the Guangzhou Science and Technology Creative Project under Key Grant No. 201604020003; the Guangdong Province Key Laboratory of Computational Science Open Grant No. 2018009; the Construction Project of Shanghai Key Laboratory of Molecular Imaging 18DZ2260400; The Natural Science Foundation of Guangdong Province, China, under Grant No. 2019A1515011168; the Clinical Research Startup Program of Southern Medical University by High-level University Construction Funding of Guangdong Provincial Department of Education under Grant No. LC2016ZD018; and the Clinical Research Program of Nanfang Hospital, Southern Medical University, under Grant Nos. 2020CR010, 2019CR002, and 2018CR040; and President’s Fund of Nanfang Hospital, Southern Medical University, under Grant No. 2019C017, in part by the Science and Technology Program of Guangzhou under Grant 201804020053, in part by Guangdong Province Key Laboratory of Computational Science at the Sun Yat-sen University under grant 2020B1212060032.

## Conflict of Interest

YLu is the founder and president of Perception Vision Medical Technologies Ltd. Co. JW is the chief technology officer and vice president of Perception Vision Medical Technologies Ltd. Co.

The remaining authors declare that the research was conducted in the absence of any commercial or financial relationships that could be construed as a potential conflict of interest.

## Publisher’s Note

All claims expressed in this article are solely those of the authors and do not necessarily represent those of their affiliated organizations, or those of the publisher, the editors and the reviewers. Any product that may be evaluated in this article, or claim that may be made by its manufacturer, is not guaranteed or endorsed by the publisher.

## Abbreviations

FFDM, full-field digital mammography; CAD, computer-aided diagnosis; ROI, region of interest; BI-RADS, Breast Imaging Reporting and Data System; CC, craniocaudal; MLO, mediolateral oblique; ReLU, rectified linear unit; MRMC, multi-reader multi-case; POM, probability of malignancy; CNN, convolutional neural network; AUC, area under the curve.

## References

[1] Smith RA Cokkinides V Brooks D Saslow D Brawley OW. Cancer Screening in the United States, 2010: A Review of Current American Cancer Society Guidelines and Issues in Cancer Screening. CA Cancer J Clin (2010) 60(2):99–119. doi: 10.3322/caac.20063 20228384

[2] Broeders M Moss S Nyström L Njor S Jonsson H Poop E. The Impact of Mammographic Screening on Breast Cancer Mortality in Europe: A Review of Observational Studies. J Med Screen (2012) 19:14–25. doi: 10.1258/jms.2012.012078 22972807

[3] Marmot MG Altman DG Cameron DA Dewar JA Thompson SG Wilcox M Independent UK Panel on Breast Cancer Screening. The Benefits and Harms of Breast Cancer Screening: An Independent Review. Lancet (2012) 380(9855):1778–86. doi: 10.1016/S0140-6736(12)61611-0 23117178

[4] Karssemeijer N Bluekens AM Beijerinck D Deurenberg JJ Beekman M Visser R. Breast Cancer Screening Results 5 Years After Introduction of Digital Mammography in a Population-Based Screening Program. Radiology (2009) 253:353–8. doi: 10.1148/radiol.2532090225 19703851

[5] Lu S Huang X Yu H Yang J Han RQ Su J. Dietary Patterns and Risk of Breast Cancer in Chinese Women: A Population-Based Case-Control Study. Lancet (2016) 388:S61. doi: 10.1016/S0140-6736(16)31988-2

[6] Rimmer A. Radiologist Shortage Leaves Patient Care at Risk, Warns Royal College. BMJ (2017) 359:j4683. doi: 10.1136/bmj.j4683 29021184

[7] Evans KK Birdwell RL Wolfe JM. If You Don’t Find it Often, You Often Don’t Find it: Why Some Cancers are Missed in Breast Cancer Screening. PLoS One (2013) 8:e64366. doi: 10.1371/journal.pone.0064366 23737980 PMC3667799

[8] Bird RE Wallace TW Yankaskas BC. Analysis of Cancers Missed at Screening Mammography. Radiology (1992) 184(3):613–7. doi: 10.1148/radiology.184.3.1509041 1509041

[9] Wong C Lim GH Gao F Jakes RW Offman J Chia KS. Mammographic Density and its Interaction With Other Breast Cancer Risk Factors in an Asian Population. Br J Cancer (2011) 104:871–4. doi: 10.1038/sj.bjc.6606085 PMC3048202 21245860

[10] El-Bastawissi AY White E Mandelson MT Taplin S. Variation in Mammographic Breast Density by Race. Ann Epidemiol (2001) 11:257–63. doi: 10.1016/s1047-2797(00)00225-8 11306344

[11] Rajaram N Mariapun S Eriksson M Tapia J Kwan PY Ho WK. Differences in Mammographic Density Between Asian and Caucasian Populations: A Comparative Analysis. Breast Cancer Res Treat (2017) 161:353–62. doi: 10.1007/s10549-016-4054-y 27864652

[12] Friedewald SM Rafferty EA Rose SL Durand MA Plecha DM Greenberg JS. Breast Cancer Screening Using Tomosynthesis in Combination With Digital Mammography. JAMA (2014) 311(24):2499–507. doi: 10.1001/jama.2014.6095 25058084

[13] Broeders MJ Onland-Moret NC Rijken HJ Hendriks JH Verbeek AL Holland R. Use of Previous Screening Mammograms to Identify Features Indicating Cases That Would Have a Possible Gain in Prognosis Following Earlier Detection. Eur J Cancer (2003) 39(12):1770–5. doi: 10.1016/s0959-8049(03)00311-3 12888373

[14] Cheng HD Shi XJ Min R Hu LM Cai XR Du HN. Approaches for Automated Detection and Classification of Masses in Mammograms. Pattern Recognit (2005) 39(4):646–68. doi: 10.1016/j.patcog.07.006

[15] Huynh BQ Li H Giger ML. Digital Mammographic Tumor Classification Using Transfer Learning From Deep Convolutional Neural Networks. J Med Imag (2016) 3(3):34501. doi: 10.1117/1.JMI.3.3.034501 PMC4992049 27610399

[16] Rodríguez-Ruiz A Krupinski E Mordang JJ Schilling K Heywang-Kobrunner SH Sechopoulos J. Detection of Breast Cancer With Mammography: Effect of an Artificial Intelligence Support System. Radiology (2019) 290(2):305–14. doi: 10.1148/radiol.2018181371 30457482

[17] Wu N Phang J Park J Shen YQ Huang Z Zorin M. Deep Neural Networks Improve Radiologists’ Performance in Breast Cancer Screening. IEEE Trans Med Imaging (2020) 39(4):1184–94. doi: 10.1109/TMI.2019.2945514 PMC7427471 31603772

[18] Jiao Z Gao X Wang Y Li J. A Deep Feature Based Framework for Breast Masses Classification. Neurocomputing (2016) 197:221–31. doi: 10.1016/j.neucom.2016.02.060

[19] Kooi T Litjens G van Ginneken B Gubern-Merida A Sancheza CI Mann R. Large Scale Deep Learning for Computer Aided Detection of Mammographic Lesions. Med Image Anal (2016) 35:303–12. doi: 10.1016/j.media.2016.07.007 27497072

[20] Simonyan K Zisserman A. Very Deep Convolutional Networks for Large-Scale Image Recognition (2015). Available at: http://arXiv.org/cs/1409.1556.

[21] Ertosun MG Rubin D. “Probabilistic Visual Search for Masses Within Mammography Images Using Deep Learning”. In: Bioinformatics and Biomedicine (BIBM). New York, NY: IEEE: IEEE International Conference (2015). p. S1310–5.

[22] Surendiran B Ramanathan P Vadivel A. Effect of BI-RADS Shape Descriptors on Breast Cancer Analysis. Int J Med Eng Inform (2015) 7(1):65–79. doi: 10.1504/IJMEI.2015.066244

[23] Sun W Tseng T-LB Zheng B Qian W. “A Preliminary Study on Breast Cancer Risk Analysis Using Deep Neural Network”. In: Breast Imaging, vol. 9699. . Cham, Switzerland: Springer International Publishing (2016). p. 385–91. Lecture Notes in Computer Science.

[24] Becker AS Marcon M Ghafoor S Wurnig MC Frauenfelder T Boss A. Deep Learning in Mammography: Diagnostic Accuracy of a Multipurpose Image Analysis Software in the Detection of Breast Cancer. Invest Radiol (2017) 52(7):434–40. doi: 10.1097/RLI.0000000000000358 28212138

[25] Agarwal R Díaz O Yap MH Lladó X Martí R. Deep Learning for Mass Detection in Full Field Digital Mammograms. Comput Biol Med (2020) 121:103774. doi: 10.1016/j.compbiomed.2020.103774 32339095

[26] Boumaraf S Liu X Ferkous C Ma X. A New Computer-Aided Diagnosis System With Modified Genetic Feature Selection for BI-RADS Classification of Breast Masses in Mammograms. BioMed Res Int (2020) 2020:7695207. doi: 10.1155/2020/7695207 32462017 PMC7238352

[27] Yan Y Conze PH Lamard M Quellec G Cochener B Coatrieux G. Towards Improved Breast Mass Detection Using Dual-View Mammogram Matching. Med Image Anal (2021) 71:102083. doi: 10.1016/j.media.2021.102083 33979759

[28] Lehman CD Wellman RD Buist DS Kerlikowske K Tosteson AN Miglioretti DL. Breast Cancer Surveillance Consortium. Diagnostic Accuracy of Digital Screening Mammography With and Without Computer-Aided Detection. JAMA Intern Med (2015) 175(11):1828–37. doi: 10.1001/jamainternmed.2015.5231 PMC4836172 26414882

[29] Lehman CD Wellman RD Buist DS Kerlikowske K Tosteson AN Miglioretti DL. Diagnostic Accuracy of Digital Screening Mammography With and Without Computer-Aided Detection. JAMA Intern Med (2015) 175:1828–37. doi: 10.1001/jamainternmed.2015.5231 PMC4836172 26414882

[30] Geras KJ Mann RM Moy L. Artificial Intelligence for Mammography and Digital Breast Tomosynthesis: Current Concepts and Future Perspectives. Radiology (2019) 293(2):246–59. doi: 10.1148/radiol.2019182627 PMC6822772 31549948

[31] Kim EK Kim HE Han K Kang BJ Sohn YM Woo OH. Applying Data-Driven Imaging Biomarker in Mammography for Breast Cancer Screening: Preliminary Study. Sci Rep (2018) 8(1):2762. doi: 10.1038/s41598-018-21215-1 29426948 PMC5807343

[32] Ha T Jung Y Kim JY Park SY Kang DK Kim TH. Comparison of the Diagnostic Performance of Abbreviated MRI and Full Diagnostic MRI Using a Computer-Aided Diagnosis (CAD) System in Patients With a Personal History of Breast Cancer: The Effect of CAD-Generated Kinetic Features on Reader Performance. Clin Radiol (2019) 74(10):817.e15–817.e21. doi: 10.1016/j.crad.2019.06.025 31362885

[33] Rodriguez-Ruiz A Lång K Gubern-Merida A Teuwen J Broeders M Gennaro G. Can We Reduce the Workload of Mammographic Screening by Automatic Identification of Normal Exams With Artificial Intelligence? A Feasibility Study. Eur Radiol (2019) 29:4825–32. doi: 10.1007/s00330-019-06186-9 PMC6682851 30993432

[34] Pinto MC Rodriguez-Ruiz A Pedersen K Hofvind S Wicklein J Kappler S. Impact of Artificial Intelligence Decision Support Using Deep Learning on Breast Cancer Screening Interpretation With Single-View Wide-Angle Digital Breast Tomosynthesis. Radiology (2021) 300(3):529–36. doi: 10.1148/radiol.2021204432 34227882

[35] van Winkel SL Rodríguez-Ruiz A Appelman L Gubern-Mérida A Karssemeijer N Teuwen J. Impact of Artificial Intelligence Support on Accuracy and Reading Time in Breast Tomosynthesis Image Interpretation: A Multi-Reader Multi-Case Study. Eur Radiol (2021) 31(11):8682–91. doi: 10.1007/s00330-021-07992-w PMC8523448 33948701

[36] Conant EF Toledano AY Periaswamy S Fotin SV Go J Boatsman JE. Improving Accuracy and Efficiency With Concurrent Use of Artificial Intelligence for Digital Breast Tomosynthesis. Radiol Artif Intell (2019) 31;1(4):e180096. doi: 10.1148/ryai.2019180096 PMC6677281 32076660

[37] Benedikt RA Boatsman JE Swann CA Kirkpatrick AD Toledano AY. Concurrent Computer-Aided Detection Improves Reading Time of Digital Breast Tomosynthesis and Maintains Interpretation Performance in a Multireader Multicase Study. AJR Am J Roentgenol (2018) 210(3):685–94. doi: 10.2214/AJR.17.18185 29064756

[38] Balleyguier C Arfi-Rouche J Levy L Toubiana PR Cohen-Scali F Toledano AY. Improving Digital Breast Tomosynthesis Reading Time: A Pilot Multi-Reader, Multi-Case Study Using Concurrent Computer-Aided Detection (CAD). Eur J Radiol (2017) 97:83–9. doi: 10.1016/j.ejrad.2017.10.014 29153373

