# Supplementary material for: Can a Computer-Aided Mass Diagnosis Model Based on Perceptive Features Learned From Quantitative Mammography Radiology Reports Improve Junior Radiologists’ Diagnosis Performance? An Observer Study
Source: Front Oncol. 2021 Dec 17;11:773389. doi: 10.3389/fonc.2021.773389 (PMC8719464; doi:10.3389/fonc.2021.773389)
Supplement: Supplementary file 1 [file DataSheet_1.docx]

**Supplementary Material**

# Appendix A: Mass characteristics by retrospective study

Table 1. the palpation of three groups

| **Palpation** |  | **training set** | **test set** | **51 cases in observer evaluation** |
| --- | --- | --- | --- | --- |
| **The activity of mass** | Removable | 66 | 3 | 17 |
|  | Semipermanent | 2 | 64 | 0 |
|  | Partially movable | 103 | 91 | 25 |
|  | Fixed | 4 | 9 | 2 |
|  | Non-palpable | 39 | 35 | 7 |
| **Hardness** | Soft | 2 | 1 | 0 |
|  | Tough | 83 | 83 | 22 |
|  | Hard | 90 | 83 | 22 |
|  | Non-palpable | 39 | 35 | 7 |
| **Growth pattern** | Expansive growth | 111 | 116 | 32 |
|  | Infiltrative growth | 64 | 51 | 12 |
|  | Non-palpable | 39 | 35 | 7 |

We set the palpation standard according to the 5^th^ BI-RADS, and each lesion was confirmed by two readers. On average, most of the masses in the training set were partially movable (103/214), and the non-palpable masses were approximated between the training set and testing set. The palpation of the mass might be different due to the radiologists’ experience and tactile sensation, and the growth pattern of the mass might also be different because of the different methods and angles of palpation.

Table 2. The change of skin and nipple

| **The change of skin and nipple** |  | **training set** | **test set** | **51 cases in observer evaluation** |
| --- | --- | --- | --- | --- |
| **Skin retraction** | Y | 24 | 18 | 4 |
|  | N | 190 | 184 | 47 |
| **Nipple retraction** | Y | 17 | 14 | 5 |
|  | N | 197 | 188 | 46 |
| **Nipple discharge** | Y | 7 | 10 | 2 |
|  | N | 207 | 192 | 49 |
| **The change of mammary areola** | Y | 1 | 2 | 1 |
|  | N | 213 | 200 | 50 |

In Table 2, on average, fewer changes in the skin and nipple were observed, training set (24/214, 17/214), and test set (18/202, 14/188). This may be because most patients underwent a detailed imaging examination when they found abnormalities during self-examination, or because lesions were found during screening, so there were relatively few changes in the skin and nipples.

Table 3. the characteristics of mass and associated features in three group

| **Main features** |  | **training set** | **test set** | **51 cases in observer evaluation** |
| --- | --- | --- | --- | --- |
| **Breast composition** | a | 6 | 5 | 3 |
|  | b | 23 | 25 | 9 |
|  | c | 169 | 154 | 35 |
|  | d | 16 | 18 | 4 |
| **Laterality** | Left | 111 | 105 | 33 |
|  | Right | 103 | 97 | 18 |
| **Quadrant** | Outer upper | 100 | 91 | 24 |
|  | Inner upper | 29 | 25 | 6 |
|  | Outer lower | 15 | 20 | 4 |
|  | Inner lower | 19 | 18 | 6 |
|  | Axillary region | 1 | 2 | 0 |
|  | Central area | 6 | 6 | 1 |
|  | Subareolar region | 11 | 13 | 4 |
|  | Other | 33 | 27 | 6 |
| **Depth** | Front | 38 | 35 | 7 |
|  | Middle | 68 | 64 | 15 |
|  | Rear | 108 | 103 | 29 |
| **Mass diameter** | d＜2 | 96 | 101 | 22 |
|  | 2≤d＜4 | 100 | 93 | 26 |
|  | d≥4 | 18 | 8 | 3 |
| **Shape** | Round | 11 | 14 | 2 |
|  | Oval | 70 | 55 | 13 |
|  | Irregular | 133 | 133 | 36 |
| **Sharpness** | Circumscribed | 55 | 54 | 11 |
|  | Obscured | 16 | 12 | 1 |
|  | Indistinct | 143 | 136 | 39 |
|  | Micolobulated | 17 | 9 | 4 |
|  | Spiculated | 70 | 52 | 12 |
| **Density** | Low | 3 | 4 | 1 |
|  | Equal | 106 | 109 | 26 |
|  | High | 104 | 87 | 23 |
|  | Fat-containing | 1 | 2 | 1 |

In Table 3, it showed that most of the cases were type C (heterogeneously dense),169/214 in training set, 154/202 in test set, 35/51 in observers’ evaluation set. Also, we can observe three signs chosen as the description of the mass lesion, which included shape, sharpness, density. Maybe most of the case’s composition of the breast was dense, and most of the masses were classified as irregular or circumscribed.

Table 4 Associated features

| **Associated features** |  | **training set** | **test set** | **51 cases in observer evaluation** |
| --- | --- | --- | --- | --- |
| **Skin retraction or thickening** | Y | 36 | 26 | 7 |
|  | N | 178 | 176 | 44 |
| **Nipple retraction** | Y | 22 | 19 | 4 |
|  | N | 192 | 183 | 47 |
| **Fat layer turbidity** | Y | 126 | 182 | 45 |
|  | N | 88 | 20 | 6 |
| **Suspensorium retraction** | Y | 24 | 12 | 4 |
|  | N | 190 | 190 | 47 |
| **Trabecular thickening** | Y | 108 | 80 | 27 |
|  | N | 106 | 122 | 24 |
| **The increase of parenchymal density** | Y | 77 | 35 | 9 |
|  | N | 137 | 167 | 42 |
| **Shape of lymph node** | Y | 26 | 23 | 4 |
|  | N | 188 | 179 | 47 |

In Table 4, we can observe that most of the associated features were negative, and the positive signs were the tissues changed nearby the masses lesion, including fat layer turbidity and trabecular thickening.

Table 5. Histopathology results

| **Variable** | Training Set (n=214) | Test Set (n=202) | 51 Cases in Observer Evaluation |
| --- | --- | --- | --- |
| **Histopathology** |  |  |  |
| Adenosis of breast | 14 | 13 | 3 |
| Basal-like breast carcinoma | 3 | 4 | 1 |
| Chronic suppurative inflammation | 2 | 2 | 1 |
| Cyst of galactostasia | 1 | 1 | 0 |
| Ductal carcinoma in situ（DICS） | 4 | 0 | 0 |
| Epidermal cyst | 0 | 1 | 0 |
| Fibroadenoma | 67 | 68 | 16 |
| Fibroadenosis | 15 | 7 | 3 |
| Fibrous adipose tissue and breast ducts | 0 | 1 | 0 |
| Granulomatous Mastitis | 0 | 2 | 0 |
| Interstitial fibers proliferate | 0 | 1 | 1 |
| Intraductal papilloma | 4 | 13 | 2 |
| Invasive Carcinoma | 2 | 1 | 0 |
| Invasive ductal carcinoma | 90 | 65 | 18 |
| Invasive lobular carcinoma | 0 | 5 | 1 |
| Leukemia | 0 | 1 | 0 |
| Mammary neuroendocrine carcinoma | 0 | 1 | 0 |
| Mixed invasive carcinoma（ILC+IDC） | 0 | 1 | 1 |
| Metaplastic breast carcinoma | 4 | 0 | 0 |
| Mucinous carcinoma | 2 | 1 | 0 |
| Papilloma | 1 | 1 | 0 |
| Phyllodes tumors | 2 | 7 | 0 |
| Pure cyst | 1 | 4 | 3 |
| Sclerosing adenosis | 1 | 0 | 0 |
| Suppurative mastitis | 1 | 1 | 0 |
| Tubular carcinoma | 0 | 1 | 1 |

In Table 5, 416 cases that met the above inclusion criteria were obtained, all of the cases accepting biopsies. In training set were 214 cases, including benign 109/214 (50.9%) and malignant 105/214 (49.1%). The test set included 202 cases (benign 122/204 (60.4%) and malignant 80/202 (39.6%) were used as independent test sets to evaluate the model.51 cases (benign 29/51 56.9% and malignant 22/51 43.1%) were randomly selected for the observer study. Most of the malignant lesions were invasive ductal carcinoma (IDC), and most of the benign lesions were Fibroadenoma.

# Appendix B: Model training and output

In the training process of stepwise regression and LDA, we do not differentiate between masses in CC-view image and masses in MLO-view image, i.e., a lesion-wise classification model is considered. In the test process, the classifier will output a malignancy probability for each mass. For a same patient, the outputs of the masses in both CC-view and MLO-view are averaged for later evaluation. The flow chart of this process is shown in Figure 1.


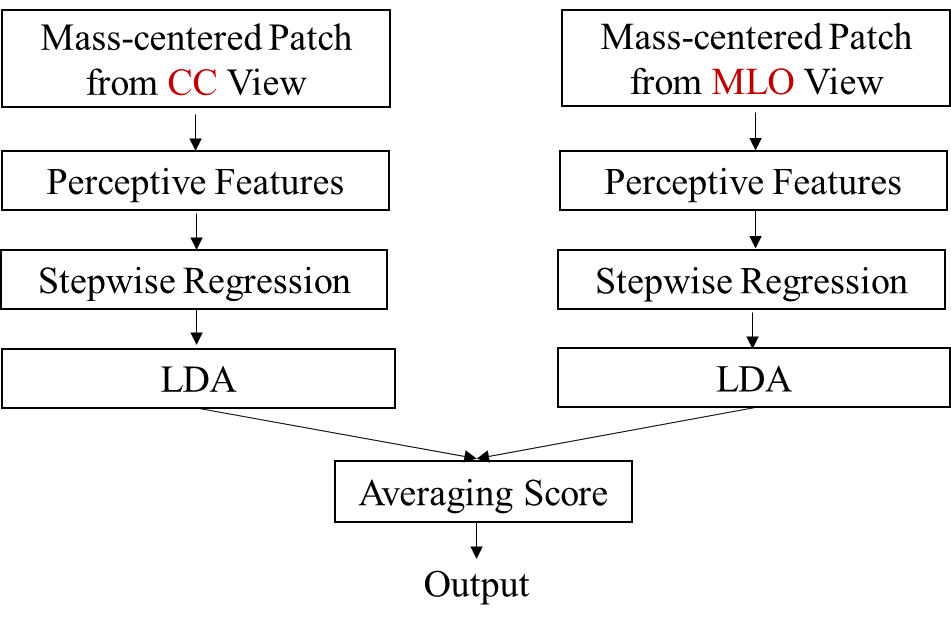


Figure 1. The flow chart of the classification system. In the test time, the outputs of CC-view and MLO-view are averaged for a same patient.

Figure 2 shows the loss curves of the first training time. The validation loss reached the lowest value and plateaued when the training reached the 70th epoch. The rest of the loss curves in other nine cross validation times were similar to these. As a result, the weights of the VGG16 network at the 70th epoch were fixed as the weights of the feature extractor.


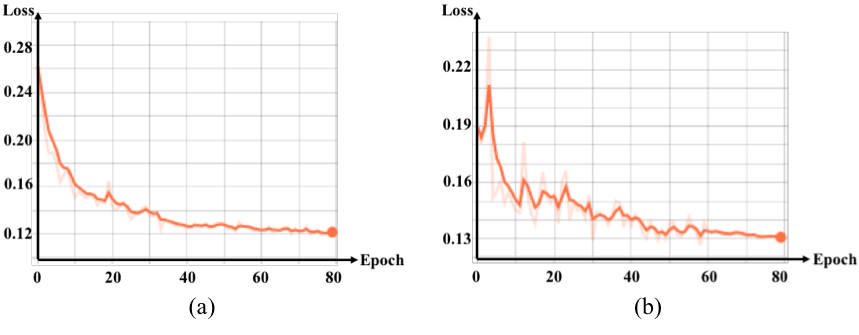


Figure 2 Loss curves. (a) The loss curve of the training set in the first time of cross validation; (b) The loss curve of the validation set in the first time of cross validation.

The vertical average ROC curve of ten-fold validation was shown in Figure 3 respectively.


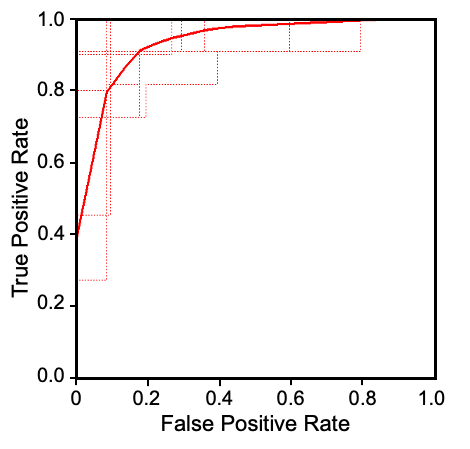


Figure 3. Vertical average ROC curve of cross validation

A malignant example and benign example are showed as following. Most of the readers changed their POM when there is the model’s support.


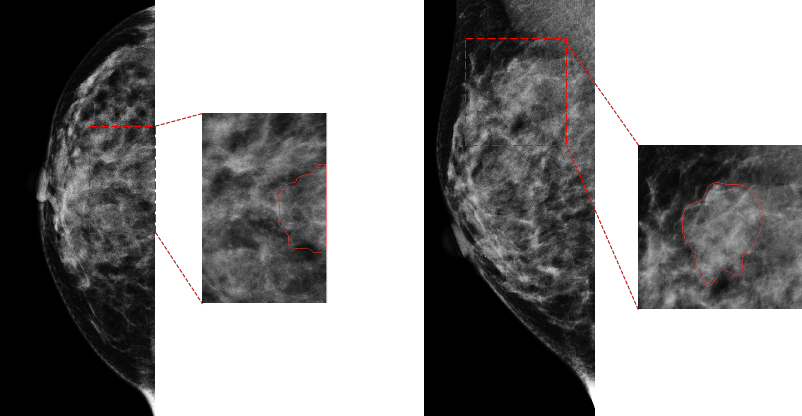


Figure 4. The CC and MLO view of the first example (ID: 11) with the mass marked by red line. The lower percentage of the POMs were classified by radiologists at the first session owing to the mass was covered by the high density of mammary glands. After aiding by CAD model, four of six radiologists had altered the POMs of the mass and classified it as a malignant mass.

Table 6. The POMs assessed by six radiologists in two sessions for the case showed in Figure 4

| Reader | w/o Reference | with Reference | Output of model | Ground-truth |
| --- | --- | --- | --- | --- |
| 1 | 30% | 30% | 78.62% | Malignant |
| 2 | 40% | 80% |  |  |
| 3 | 10% | 70% |  |  |
| 4 | 1% | 80% |  |  |
| 5 | 30% | 50% |  |  |
| 6 | 10% | 5% |  |  |


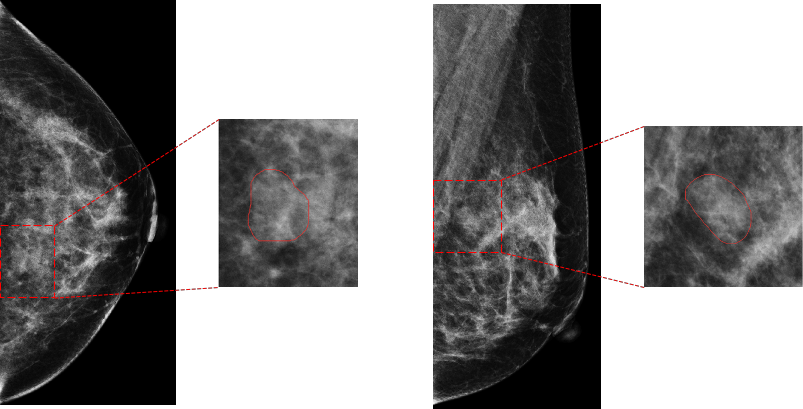


Figure 5. The CC and MLO view of the second example (ID: 44) with the mass marked by red line. In first session, we found that the mass was overrated by the junior radiologists’ group which both of POMs were approximately 50% malignant.

Table 6 The POMs assessed by six radiologists in two sessions for the case showed in Figure 8

| Reader | w/o Reference | with Reference | Output of model | Ground-truth |
| --- | --- | --- | --- | --- |
| 1 | 40% | 10% | 3.06% | Benign |
| 2 | 55% | 6% |  |  |
| 3 | 3% | 2% |  |  |
| 4 | 10% | 40% |  |  |
| 5 | 2% | 1% |  |  |
| 6 | 0% | 0% |  |  |

# Appendix C:

# The table used by readers to record the reading results in the multi-reader multi-case observer study

Table 7. The table that records reader’s assessment in multi-reader multi-case study

|  | | |
| --- | --- | --- |
| FFDM evaluation（the most suspicious lesion） | | |
| Breast composition: a □ b □ c □ d □ | | |
| Laterality | right □ | left □ |
| Quadrant（multiple choices） | Outer upper □ Inner upper □  Outer lower □ Inner lower □  Axillary region □ Central area □  Subareolar region □ Other | Outer upper □ Inner upper □  Outer lower □ Inner lower □  Axillary region □ Central area □  Subareolar region □ Other |
| Mass feature | | |
| Shape | Round□ Oval□ Irregular□ | Round□ Oval□ Irregular□ |
| Sharpness（④, ⑤multiple choices，①，②，③single choices） | ①Circumscribed□ ②Obscured□ ③Indistinct□  ④Micolobulated□ ⑤Spiculated□ | ①Circumscribed□ ②Obscured□ ③Indistinct□  ④Micolobulated□ ⑤Spiculated□ |
| Density | Low□ Equal□ High□ Fat-containing□ | Low□ Equal□ High□ Fat-containing□ |
| BI-RADS category | 0□ 1□ 2□ 3□ 4a□ 4b□ 4c□ 5□ | 0□ 1□ 2□ 3□ 4a□ 4b□ 4c□ 5□ |
| benign or malignancy classification（POM:0-100%） | benign □ malignancy □  Possibility of Malignancy [POM] % | benign □ malignancy □  Possibility of Malignancy [POM] % |
| diagnosis time[min/s] | min s | min s |

# Specific BI-RADS category changes in the multi-reader multi-case study.

In MRMC evaluation, all the readers adjusted BI-RADS category of partial cases with model reference, which focused on BI-RADS 2,3,4. The assessments trended toward increase in BI-RADS 4, and less cases were defined as BI-RADS 2 or 3 with the model reference. The BI-RADS category changes between the two study sessions were shown in Table 8, 9, 10 and 11.

Table 8. BI-RADS category from report

| **BI-RADS category** | **training set** | **test set** | **51 cases in observer evaluation** | **Totally** |
| --- | --- | --- | --- | --- |
| **0** | 2 | 3 | 0 | 5 |
| **1** | 0 | 0 | 0 | 0 |
| **2** | 17 | 26 | 4 | 43 |
| **3** | 25 | 22 | 5 | 47 |
| **4a** | 53 | 53 | 11 | 106 |
| **4b** | 22 | 24 | 10 | 46 |
| **4c** | 23 | 28 | 8 | 51 |
| **5** | 62 | 42 | 13 | 104 |
| **6** | 10 | 4 | 0 | 14 |

In table 8, we can see the categories extracted by the readers, which were assessed by the radiologists from mammography. Most of the masses were assessed as BI-RADS 4 (totally 215). Because of dense tissue occlusion, radiologists might improve the assessment of the lesion. As we all known, radiologists evaluated masses as BI-RADS 4, which considered those lesions were uncertain malignant or suspicious malignant for more biopsies to confirm the histopathological results. At this time, we might consider the radiologists overestimated the lesions, which were more pronounced in the junior group (reader-1 42/51, reader-2 34/51).

**Table 9.** The BI-RADS category assessment without reference of our model

| **BI-RADS** | **reader 1** | **reader 2** | **reader 3** | **reader 4** | **reader 5** | **reader 6** |
| --- | --- | --- | --- | --- | --- | --- |
| **0** | 0 | 0 | 0 | 0 | 0 | 0 |
| **1** | 0 | 0 | 0 | 0 | 0 | 0 |
| **2** | 0 | 1 | 1 | 15 | 2 | 4 |
| **3** | 4 | 1 | 7 | 1 | 13 | 12 |
| **4** | 43 | 34 | 35 | 25 | 31 | 25 |
| **4a** | 16 | 6 | 13 | 8 | 17 | 13 |
| **4b** | 21 | 18 | 12 | 9 | 8 | 8 |
| **4c** | 6 | 10 | 10 | 8 | 6 | 4 |
| **5** | 4 | 15 | 8 | 10 | 5 | 10 |
| **6** | 0 | 0 | 0 | 0 | 0 | 0 |

**Table 10.** The BI-RADS category assessment with reference of our model

| **BI-RADS** | **reader 1** | **reader 2** | **reader 3** | **reader 4** | **reader 5** | **reader 6** |
| --- | --- | --- | --- | --- | --- | --- |
| **0** | 0 | 0 | 0 | 0 | 0 | 0 |
| **1** | 0 | 0 | 0 | 1 | 0 | 0 |
| **2** | 0 | 3 | 0 | 7 | 2 | 3 |
| **3** | 3 | 0 | 6 | 3 | 6 | 13 |
| **4** | 43 | 36 | 37 | 28 | 34 | 26 |
| **4a** | 13 | 8 | 17 | 8 | 13 | 14 |
| **4b** | 22 | 17 | 12 | 11 | 13 | 7 |
| **4c** | 8 | 11 | 8 | 9 | 8 | 5 |
| **5** | 5 | 12 | 8 | 12 | 9 | 9 |
| **6** | 0 | 0 | 0 | 0 | 0 | 0 |

**Table 11.** Specific BI-RADS changes under supporting by CAD model in multi-reader multi-case study

| **Readers** | **BI-RADS** | **1** | **2** | **3** | **4** | **5** | **6** | **Total** |
| --- | --- | --- | --- | --- | --- | --- | --- | --- |
| **Increase** | **2→3** | 0 | 0 | 1 | 2 | 0 | 1 | 4 |
|  | **2→4a** | 0 | 1 | 0 | 4 | 1 | 0 | 6 |
|  | **2→4c** | 0 | 0 | 0 | 1 | 0 | 0 | 1 |
|  | **3→4a** | 3 | 0 | 2 | 0 | 3 | 2 | 10 |
|  | **3→4b** | 0 | 0 | 1 | 0 | 3 | 1 | 5 |
|  | **3→4c** | 0 | 0 | 0 | 1 | 0 | 0 | 1 |
|  | **3→5** | 0 | 0 | 0 | 0 | 1 | 0 | 1 |
|  | **4a→4b** | 7 | 2 | 2 | 4 | 6 | 0 | 21 |
|  | **4a→4c** | 0 | 0 | 1 | 0 | 2 | 0 | 3 |
|  | **4b→4c** | 3 | 5 | 3 | 2 | 3 | 0 | 16 |
|  | **4b→5** | 0 | 0 | 0 | 0 | 1 | 0 | 1 |
|  | **4c→5** | 1 | 3 | 1 | 3 | 2 | 1 | 11 |
|  | **5→4c** | 0 | 3 | 1 | 1 | 0 | 2 | 7 |
| **Decrease** | **5→4b** | 0 | 3 | 0 | 0 | 0 | 0 | 3 |
|  | **4c→4b** | 0 | 3 | 3 | 1 | 1 | 0 | 8 |
|  | **4c→4a** | 0 | 1 | 3 | 0 | 0 | 0 | 4 |
|  | **4b→4a** | 2 | 2 | 5 | 1 | 1 | 2 | 13 |
|  | **4b→3** | 1 | 0 | 0 | 0 | 0 | 0 | 1 |
|  | **4b→2** | 0 | 2 | 0 | 0 | 0 | 0 | 2 |
|  | **4a→3** | 1 | 0 | 1 | 1 | 1 | 3 | 7 |
|  | **3→2** | 0 | 1 | 0 | 0 | 1 | 0 | 2 |
|  | **2→1** | 0 | 0 | 0 | 1 | 0 | 0 | 1 |

The BI-RADS category changed after support by the CAD model. Each reader adjusted BI-RADS as increasing or decreasing, which each reader adjusted closed. In this form, we found that the most increasing BI-RADS were BI-RADS 4 [4a to 4b], and the most decreasing were BI-RADS 4b to 4a.

We believed that the change in BI-RADS was due to the fact that radiologists had a more positive understanding of the location and sharpness of the mass after using CAD supported (CAD could improve the location and sharpness of the mass), and decreased or increased the level of some uncertain or suspicious signs, resulting in a change in BI-RADS classification, which was more obvious in junior radiologists.

**Appendix D: Table that records reader’s assessment in stand-alone study**

| case number | name | age | Breast composition | Laterality | Quadrant | benign or malignancy | diagnosis | BI-RADS category | approve of the BI-RADS results or not（√ or ×） | Possibility of Malignarcy (POM)（0-100%） |
| --- | --- | --- | --- | --- | --- | --- | --- | --- | --- | --- |
| 157 | XXX | 39 | c | left | outer upper | benign | Fibroadenoma | 3 |  |  |
| 218 | XXX | 42 | c | left | upper | benign | Fibroadenosis | 2 |  |  |
| 220 | XXX | 31 | d | left | outer upper | benign | none | 0 |  |  |
| 225 | XXX | 52 | c | left | outer upper | benign | none | 4b |  |  |
| 226 | XXX | 45 | c | left | other | benign | Fibroadenoma | 3 |  |  |
| 250 | XXX | 51 | c | right | outer upper | malignancy | none | 4b |  |  |
| …… | | | | | | | | | | |
| 252 | XXX | 46 | c | right | outer upper | malignancy | none | 4c |  |  |
| 253 | XXX | 36 | c | right | outer upper | benign | Fibroadenoma | 4a |  |  |
| 339 | XXX | 55 | c | left | central area | benign | Pure cyst | 2 |  |  |
| 385 | XXX | 48 | c | left | upper | malignancy | none | 4c |  |  |
| 389 | XXX | 67 | c | right | inner upper | malignancy | none | 4c |  |  |
| 390 | XXX | 49 | b | left | outer upper | malignancy | none | 5 |  |  |

Stand-along evaluation form was filled by reading 7 and 8 after comparing the reported results of the initial diagnosis with the results of BI-RADS classification: If radiologists approved the initial result, checked yes in the tables, and give the percentage of malignant probability of the mass (%). If radiologists did not approve the initial result, they filled in the new BI-RADS classification and gave the new percentage of malignant probability (%).
